# Analytical Performance and Concordance with Next-Generation Sequencing of a Rapid, Multiplexed dPCR Panel for the Detection of DNA and RNA Biomarkers in Non-Small-Cell Lung Cancer

**DOI:** 10.3390/diagnostics13213299

**Published:** 2023-10-25

**Authors:** Kerri Cabrera, Jeffrey Gole, Bryan Leatham, Matthew J. Springer, Molly Smith, Leah Herdt, Lucien Jacky, Bradley A. Brown

**Affiliations:** ChromaCode Inc., 2330 Faraday Ave., Carlsbad, CA 92008, USA; jgole@chromacode.com (J.G.); mspringer@chromacode.com (M.J.S.); msmith@chromacode.com (M.S.); lherdt@chromacode.com (L.H.); ljacky@chromacode.com (L.J.);

**Keywords:** multiplex, digital PCR, NSCLC

## Abstract

FDA approval of targeted therapies for lung cancer has significantly improved patient survival rates. Despite these improvements, barriers to timely access to biomarker information, such as nucleic acid input, still exist. Here, we report the analytical performance and concordance with next-generation sequencing (NGS) of a highly multiplexed research-use-only (RUO) panel using digital PCR (dPCR). The panel’s analytical sensitivity and reactivity were determined using contrived DNA and RNA mixes. The limit of blank was established by testing FFPE curls classified as negative by pathology. Concordance was established on 77 FFPE samples previously characterized using the Oncomine Precision Assay^®^, and any discordant results were resolved with Archer Fusionplex^®^ and Variantplex^®^ panels. The analytical sensitivity, reported as the estimated mutant allele fraction (MAF), for DNA targets ranged from 0.1 to 0.9%. For RNA targets (ALK, RET, ROS, NTRK 1/2/3 Fusions, and MET Exon 14 skipping alteration), the analytical sensitivity ranged from 23 to 101 detected counts with 5 ng of total RNA input. The population prevalence-based coverage ranged from 89.2% to 100.0% across targets and exceeded 99.0% in aggregate. The assay demonstrated >97% concordance with respect to the comparator method.

## 1. Introduction

An estimated 236,740 lung and bronchus cancer cases were reported in the United States in 2022, with a five-year relative survival rate of approximately 23% [1]. In the last five years, targeted therapies have increased three-year survival rates by 2.3–13.7% [2,3,4]. As of the publication of this article, with 32 FDA-approved targeted therapies, non-small-cell lung cancer is the solid tumor type with the most approved therapies targeting driver mutations [5,6,7,8,9]. Despite these advances, recent studies indicate that for individuals with NSCLC, equity and accessibility are the main limitations of the use of targeted therapies [2,6,7,10]. A study leveraging a database with commercial and Medicare claims from over 500,000 NSCLC patients in the United States reported that approximately 50% of patients do not obtain complete biomarker testing [6,7]. Of the patients that did receive biomarker test results, 29% did not get the appropriate targeted therapy [6,7]. The top reasons cited by oncologists for not testing all lung cancer patients include tissue or sample limitations, cost of and access to testing, long turnarounds for sequential gene testing, or having to send out for testing [5,11,12,13]. One way to approach this problem would be to increase the timely availability of rapid biomarker testing performed locally in the hospital setting.

Two common molecular biomarker testing modalities include next-generation sequencing (NGS) and sequential single-gene PCR. While NGS provides sequence information of entire genes and regions of the genome, enabling comprehensive detection of multiple variants when present, there are also several critical challenges with sequencing-based approaches. One key challenge is time from sample collection to reported result; 13.1% of NGS testing had a turnaround time (TAT) greater than 14 days, which exceeds the TAT guideline established by the College of American Pathologists, International Association for the Study of Lung Cancer (IASLC), and the Association for Molecular Pathology [7]. Extensive turnaround times additionally have an outsized impact on late-stage patients who have more aggressive cancers and speed to result for biomarker testing should be addressed [3]. In addition to lengthy TAT, approximately 22% of patients do not receive NGS results because of an insufficient quantity of sample or poor sample quality [7,14]. NGS assays feature a high-complexity workflow and analysis that return many variants, some of which are not clinically actionable. In sharp contrast, single-gene PCR tests are manageable in terms of complexity, cost, actionable marker detection, and turnaround time. However, sample sufficiency remains a hurdle due to the need to split the sample across many tests or wells to get a complete result [15,16].

This article describes a highly multiplexed digital PCR (dPCR) assay that reports results for 15 relevant NSCLC variants in nine genes using amplitude modulation and multi-spectral encoding [17]. The panel is designed to detect clinically actionable variants in NSCLC, is compatible with formalin-fixed paraffin-embedded (FFPE) tissue specimens and utilizes a low mass input that could rescue many samples that NGS workflows designate as quantity not sufficient (QNS) [18,19,20,21,22,23,24]. Using dPCR for the panel reduces the complexity of the workflow and TAT by decreasing the number of user manipulations compared to NGS-based workflows. The HDPCR NSCLC panel uses a simplified workflow involving only two touch points post extraction, thus enabling a TAT of less than four hours, excluding extraction time. Cloud-based analysis simplifies and accelerates results interpretation, allowing for results generation in less than 24 h. Additionally, testing for multiple actionable biomarkers in a single test potentially improves tissue requirements compared to single-gene testing. The panel performance reported here includes analytical sensitivity, analytical reactivity (inclusivity), and method correlation with currently accepted amplicon based NGS methodologies.

## 2. Materials and Methods

### 2.1. Materials

Plasmids containing the target sequences were sourced from IDT (San Diego, CA, USA) and are detailed in Supplementary Table S1. De-identified, remnant human biological FFPE specimens from NSCLC patients were obtained from Precision for Medicine (Frederick, MD, USA), BioChain (Newark, CA, USA), or CHTN (Durham, NC, USA). Specimen details are listed in Supplementary Table S2. Oncomine Precision Assay testing was performed by Precision for Medicine, and results were reported for all positive specimens. Negative specimens were also tested and comprised of non-tumor adjacent tissue. The mean age of the individual at time of sample acquisition was 63.5 years (standard deviation of 11.1).

### 2.2. HDPCR NSCLC

High Definition (HDPCR) NSCLC panel utilizes dPCR, where endpoint fluorescent intensities are modulated so that each unique target produces a unique endpoint intensity [17,25,26]. The HDPCR NSCLC Panel (ChromaCode, Carlsbad, CA, USA) comprises three wells: two wells to detect DNA targets, and one to detect RNA fusions. All runs were performed on QIAcuity (Qiagen, Hilden, Germany) using the QIAcuity Nanoplate 26K 24-well plate. The mastermix for DNA wells was formulated by combining 10.5 µL of QIAcuity Probe Master Mix, 8.4 µL of HDPCR Mix, and 2.1 µL of molecular grade water per reaction. The master mix for each RNA well was formulated by combining 10.5 µL of QIAcuity OneStep Advance Probe Master Mix (Qiagen, Germany), 0.45 µL of OneStep RT Mix (Qiagen, Germany), 8.4 µL of HDPCR Mix, and 1.65 µL of molecular grade water per reaction. After the preparation of each mastermix, 21 µL of the sample was combined with 21 µL of the appropriate mastermix and mixed thoroughly. From this reaction mixture, 39 µL was loaded into a well on the QIAcuity Nanoplate. The plate then underwent thermocycling and signal detection on the QIAcuity according to the instructions for use, as follows: (1) reverse transcription at 50 °C for 40 min, (2) preheat to 95 °C for two minutes, (3) 35 cycles consisting of denaturing (95 °C, 30 s) followed by annealing/extension (58 °C for one minute). Terminal fluorescence intensity data were collected in all five available color channels. Analysis was performed using ChromaCode Cloud, which reports detected targets, estimated MAF for DNA targets and positive partition count for RNA targets. The estimated MAF is calculated as (target counts/IC counts) × 100.

### 2.3. Analytical Sensitivity

Negative FFPE background was prepared by extracting DNA and RNA from pathology-negative FFPE samples using the Maxwell HT FFPE DNA Isolation System according to the recommended protocol for DNA and RNA extraction from a single FFPE sample. The limit of detection for DNA targets was established by spiking plasmids containing the target sequences into negative FFPE background at various MAF concentrations. The RNA fusion targets were transcribed from plasmids using the HiScribe T7 High Yield RNA Synthesis Kit (NEB, Ipswich, MA, USA), isolated using the Monarch Kit (NEB, Ipswich, MA, USA) and spiked into negative FFPE RNA background.

The limit of detection for DNA targets was established at two input amounts: 20 ng and 7.5 ng of DNA per well (40 ng and 15 ng in total for both DNA wells). Mutant allele fractions (MAF) of 0.5%, 1%, 2%, 4%, and 10% were tested at both the 20 ng and 7.5 ng total input amounts. The RNA fusion targets were tested at 5 ng total RNA input. Range-finding was conducted by testing 25 total replicates in a series of five decreasing dilutions. For each target, the lowest concentration at which all five replicates were positive during range-finding was further evaluated with 20 replicates. The limit of detection is reported at the lowest concentration, where greater than 18/20 replicates were detected.

### 2.4. MAF Correlation Study

Negative FFPE background (screened with HDPCR NSCLC assay and confirmed negative for relevant panel targets) and EGFR G719S positive samples were prepared by extracting DNA from Pan-Cancer 6-Fusion Panel FFPE (Horizon Discovery, Cambridge, UK) and 50% EGFR G719S FFPE DNA (Horizon Discovery, Cambridge, UK) using the Maxwell HT FFPE DNA Isolation System (Promega, Madison, WI). Samples of varying mutant allele fractions (2%, 5%, 10%, 20%, and 40%) were prepared by spiking in 50% EGFR G719S FFPE into negative FFPE background at various MAF concentrations. Samples were analyzed with the HDPCR NSCLC panel at 5 ng, 10 ng, 20 ng, and 40 ng input.

### 2.5. Analytical Inclusivity

In silico analysis of designs was performed using the COSMIC Mutation Database v97 [27]. The prevalence of each required target assay within the total population of somatic variants at each locus in non-small-cell lung carcinomas (NSCLC) was determined. The parameters utilized in filtering the data are recorded in Table S3. Post filtering, prevalence was calculated based on the count number of distinct entries in the “sample name field”. In silico analysis was completed by confirming the % homology with primer and probe sequences and/or evaluating the binding kinetics of the assay to variant or wild-type sequences. From the in silico results, variants of indeterminant result or variants of high prevalence were then empirically tested.

Analytical inclusivity of the HDPCR NSCLC panel was established by spiking quantified plasmids containing the different target sequences into the appropriate negative FFPE background (RNA or DNA, depending on the well). Each plasmid was tested at 3–5 times the established limit of detection for that target, in two replicates. If a replicate was not detected, the plasmid was tested at a concentration 10 times higher. If no replicate was detected at this higher concentration, the assay was determined to be non-inclusive for that specific sequence. Prevalence was estimated from the reported occurrences of unique sample IDs for each COSMIC ID (LEGACY_MUTATION_ID) associated with a reportable in the filtered COSMIC Mutation Database v97 downloaded on 08FEB2023, as outlined in Table S3.

### 2.6. Limit of Blank

Sixty unique FFPE curls from 29 unique blocks classified as negative by pathology were tested to evaluate limit of blank. DNA and RNA were extracted from a single 10 µm curl using the Maxwell HT FFPE DNA Isolation System (Promega, Madison, WI, USA) on a KingFisher™ Flex instrument (Thermofisher, Carlsbad, CA, USA). Subsequent to extraction, eluates were quantified using either the Qubit dsDNA BR Assay Kit (Invitrogen, Waltham MA, USA) or the Qubit RNA BR Assay Kit (Invitrogen, Waltham, MA, USA). Samples were evaluated with the HDPCR NSCLC panel in accordance with the previously mentioned methodologies. If a target was detected with the HDPCR NSCLC panel, the sample in question was sent for discordant resolution with the Variant Plex solid tumor focus (ArcherDx, Boulder, CO, USA) or the Fusion Plex Lung (ArcherDx, Boulder, CO, USA).

### 2.7. Concordance Study

A total of 77 unique FFPE blocks (77 positive samples, as determined by Oncomine Precision Assay) from lung tissue were enrolled in the concordance study. DNA and RNA were extracted from a single 10 µm curl using the Maxwell HT FFPE DNA Isolation System (Promega, Madison, WI, USA) on the KingFisher™ Flex instrument (Thermofisher, Carlsbad, CA). Following extraction, eluates were quantified using either the Qubit dsDNA BR Assay Kit (Invitrogen, Waltham, MA, USA) or the Qubit RNA BR Assay Kit (Invitrogen, Waltham, MA, USA). Samples were evaluated with the HDPCR NSCLC panel according to the above-mentioned methodologies. Results from the Oncomine Precision Assay (from separate sections of the same block) and the HDPCR NSCLC panel were compared, and any discordant samples (same section as evaluated by HDPCR) were sent for discordant resolution using the Variant Plex solid tumor focus (ArcherDx, Boulder, CO, USA) or the Fusion Plex Lung (ArcherDx, Boulder, CO, USA).

## 3. Results

### 3.1. Analytical Sensitivity, Limit of Detection (LOD)

Analytical sensitivity was reported in estimated mutant allele fractions (MAF) for DNA targets and in positive partition counts for RNA targets. Each assay featured an internal control (IC) to determine if sufficient amplifiable nucleic acid had been loaded into the well. The estimated MAF for DNA targets was highly correlated (r^2^ > 0.8) but was affected by the ng input of (Figures S1 and S2). At 20 ng input (1854 average IC Counts) of DNA per well, the LOD ranged from 0.8% to 4.9% estimated MAF (Table 1). When the total DNA input was decreased to 7.5 ng per well (476 average IC Counts), the LOD ranged from 2.4% to 10.9% MAF (Table 1). With an input of 5 ng (97 average IC counts), the LOD for RNA targets ranged from 23 to 101 counts (Table 2). These results indicate that even with minimal inputs of DNA and RNA, the HDPCR NSCLC panel is sensitive to all targets.

### 3.2. Analytical Inclusivity

Analytical inclusivity was evaluated both in silico for DNA targets and empirically for DNA and RNA fusion targets, by testing plasmids spiked in an FFPE negative matrix. In silico analysis identified 31 DNA targets for subsequent empirical evaluation, while all RNA fusion targets were evaluated empirically. The population prevalence-based coverage for EGFR Exon 20 Insertions, EGFR Exon 20 deletions, ERBB2 Exon 20 Insertions, and EGFR G719X variants was determined to be 89.2%, 99.5%, 99.4%, and 100%, respectively. These results are detailed in Table 3 and Table S4. All RNA fusion targets were evaluated empirically, using 96 different variations. The population prevalence-based coverage for ALK, ROS1, RET and NTRK fusions was determined to be 99.0%, 100%, 98.7%, and 95.1%, respectively. The results are detailed in Table 4 and Table S4.

### 3.3. Limit of Blank (LoB)

The HDPCR panel was used to evaluate 60 unique FFPE curls from 29 unique blocks, all of which were classified as negative by pathology. Of the 60 samples run, all passed QC metrics and gave valid results, resulting in a 100.0% (94.0–100.0%, 60/60) sample validity rate. Fifty-eight DNA samples were accurately identified as true negatives (TNs), while two DNA samples were called false positives (FPs). The DNA samples had a total NPA of 96.7% (88.6–99.1%). Fifty-nine RNA samples were called true negatives (TNs), one sample was called false positive (FP). The RNA samples had a total NPA of 98.3% (88.6–99.1%).

### 3.4. Concordance Study

The HDPCR NSCLC panel was used to evaluate 77 unique clinical FFPE samples obtained from Precision for Medicine (Frederick, MD, USA), BioChain (Newark, CA, USA), or CHTN (Durham, NC, USA), which were known to harbor clinically relevant mutations that can be detected with the HDPCR assay. This study functioned as a clinical validation of our method in comparison to orthogonal testing strategies (NGS). The internal control (IC) in DNA wells failed in seventeen of the seventy-seven samples, while the RNA IC failed in six of the seventy-seven samples. These IC failures were found to correlate with the source vendors of the samples, showing variability ranging from 0% to 63%, depending on the specific vendor (Table S5).

In samples that passed IC quality criteria (QC), the concordance of the HDPCR NSCLC panel with the comparator method was 97.3%. The positive percent agreement (PPA) for individual targets ranged from 60.0–100.0% while the positive predictive value (PPV) ranged from 62.5% to 100.0%, prior to discordant resolution. Low PPA/PPV values are driven by the minimal number of positives samples available for certain targets and the high level of discordant results. The negative percent agreement (NPA) ranged from 95.0 to 100.0% and the negative predicted value (NPV) ranged from 94.4 to 100.0% (Table 5). Discordant samples were evaluated from the same extracted material whenever possible and with either the VariantPlex solid tumor focus panel for DNA targets or the FusionPlex Lung for RNA targets. After discordant resolution, each target’s PPA ranged from 71.4 to 100.0% and PPV ranged from 88.9 to 100.0%. The NPA ranged from 98.1 to 100.0% and the NPV ranged from 97.0 to 100.0% for individual targets after discordant resolution.

In total, there were 27 discordant results with the comparator method. Discordant resolution aligned with the HDPCR NSCLC panel in 77.8% (21/27) of discordant samples and aligned with the comparator in 22.2% (6/27) of samples. Of the six discordant results that aligned with the comparator method, 50.0% (3/6) of the results were novel fusions that are outside the inclusivity of the HDPCR NSCLC Panel, leaving 3/6 incongruous samples remaining. A comprehensive breakdown of all discordant analysis outcomes is detailed in Table 6 and Table S6.

## 4. Discussion

The HDPCR NSCLC panel uses a simplified workflow involving only two touch points post extraction on dPCR, allowing a TAT of less than 24 h. The results from the studies presented here illustrate that the HDPCR NSCLC panel, utilizing dPCR, achieved a sensitivity down to 0.8% estimated MAF for DNA targets and down to 23 positive partition counts for RNA targets, and an accuracy greater than 99% with comparator results after discordant resolution.

Here, we demonstrate an estimated MAF limit of detection between 2.4 and 10.9% when utilizing as little as 15 ng of DNA input split across two wells (7.5 ng per well). At a 40 ng (20 ng per well) DNA input amount, we report an estimated MAF limit of detection between 0.8 and 4.9%. In the future, the HDPCR NSCLC panel can be evaluated with cfDNA and cfRNA to potentially provide a more sensitive and straightforward workflow for liquid biopsy applications.

One disadvantage of traditional PCR approaches in detecting variants is poor sequence coverage or inclusivity [28]. The HDPCR NSCLC panel was designed for high inclusivity of highly variable targets like EGFR Exon 20 insertions (89%), EGFR Exon 19 deletions (99%), and RNA fusions (95–100%). The high inclusivity provides increased confidence in negative results.

The HDPCR NSCLC panel demonstrated high concordance (>97%) with the comparator methods before discordant resolution, and 77.8% (21/27) of discordant results were resolved in favor of HDPCR. Taken together, these results demonstrate how the HDPCR NSCLC panel can test for actionable variants with low nucleic acid input using a simple PCR-based workflow. For the RNA fusion targets, the primary source of false negatives, five of nine, was the detection of novel fusions that were not within the scope of the HDPCR panel.

The ability to provide faster, more accurate results for actionable biomarkers is a crucial step toward democratizing testing. Here, we present a dPCR panel that provides a coverage of actionable biomarkers with high concordance with NGS. The simplified workflow and analysis make it a potential solution for improving accessibility to biomarker testing.

## Figures and Tables

**Table 1:** Limit of detection for DNA targets. The limit of detection reported in estimated MAF as determined by ChromaCode Cloud 5.0 software. Input amount is defined in ng for each well as measured by Qubit. Mean counts represent the average number of positive partitions at the reported limit of detection. The detection rate is the positive results out of 20 total replicates.

	7.5 ng Input			20 ng Input		
Target	Mean Counts	Detection Rate	Estimated MAF	Mean Counts	Detection Rate	Estimated MAF
EGFR Exon 19 deletions	76.7	20/20	10.9%	30.2	20/20	1.2%
EGFR G719X	40.9	20/20	6.3%	33.8	20/20	2.7%
EGFR S768I	45.5	20/20	9.6%	63.9	20/20	3.2%
ERBB2 insertions	41.6	20/20	7.4%	32.2	20/20	2.4%
EGFR L858R	70.8	20/20	8.8%	58.4	19/20	2.6%
EGFR Exon 20 insertions	60.4	20/20	10.1%	58.8	20/20	4.9%
BRAF V600E	21.0	20/20	2.4%	44.3	20/20	2.1%
KRAS G12C	23.1	20/20	3.6%	34.1	20/20	2.3%
EGFR T790M	37.2	20/20	9.5%	19.3	20/20	0.8%
EGFR L861Q	40.4	20/20	8.3%	39.9	20/20	3.4%

**Table 2:** Limit of detection for RNA Targets. The limit of detection reported mean counts by ChromaCode Cloud 5.0 software. Input amount is defined in ng for each well as measured by Qubit. Mean counts represent the average number of positive partitions at the reported limit of detection. The detection rate is the positive results out of 20 total replicates.

	5 ng Input	
Target	Mean Counts	Detection Rate
ALK fusions	23	21/21 ^1^
RET fusions	23	19/19 ^2^
ROS1 fusions	72	19/20
MET Exon 14 skipping mutations	101	20/20
NTRK 1/2/3 fusions	85	20/20

^1^ 21 total replicates were run for ALK fusions due to operator error, all replicates gave valid results. ^2^ 20 total replicates were run for RET fusions. One replicate failed because of low IC and was excluded from the final analysis.

**Table 3:** Inclusivity results for DNA targets. In silico and empirical (bench testing) results for inclusivity by target. Unique variants listed in the COSMIC Mutation Database for each target were tested via in silico analysis. Any variants of high prevalence or those with indeterminant binding kinetics during in silico analysis were also evaluated empirically. Overall Inclusivity refers to the percent of variants reported in the COSMIC Mutation Database detected by the NSCLC HDPCR panel. Positive Patients per 1000 is the number of samples predicted to be positive for this target based on the reported prevalence in the COSMIC database per 1000 NSCLC samples. Missed Calls due to Inclusivity details the number of results impacted per 1000, due to variants outside the scope of the NSCLC panel.

Target	Overall Inclusivity (Prevalence Weighted)	Positive Samples per 1000 NSCLC Samples	Missed Calls per 1000 NSCLC Samples
EGFR Exon 20 Insertions	89.2%	9	1
EGFR Exon 19 Deletions	99.5%	131	0.7
EGFR G719X	100%	10	0
ERBB2	99.4%	9	<0.1

**Table 4:** Inclusivity results for RNA Targets. Empirical (bench testing) results for inclusivity by target. Overall Inclusivity refers to the percentage of variants reported in the COSMIC Mutation Database detected by the NSCLC HDPCR panel. Positive Patients per 1000 is the number of patients predicted to be positive for this target based on the reported prevalence in the COSMIC database per 1000 NSCLC samples. Missed Calls due to Inclusivity details the number of patients impacted per 1000 due to variants outside the scope of the NSCLC panel.

Target	Overall Inclusivity (Prevalence Weighted)	Positive Patients Per 1000 NSCLC Samples	Missed Calls Per 1000 NSCLC Samples
ALK	99%	27	<0.3
ROS1	100%	4	0
RET	98.7%	4	<0.1
NTRK	95.1%	0	0

**Table 5:** Concordance data reported by target for Oncomine Precision Assay and HDPCR NSCLC panel. True positive (TP), true negative (TN), false positive (FP), positive percent agreement (PPA), positive predictive value (PPV), negative predictive value (NPV) and negative percent agreement (NPA). Adjusted values after discordant resolution are placed in parenthesis.

Target	TP	TN	FP	FN	PPA (PPA Post Discordant)	NPA (NPA Post Discordant)	PPV (PPV Post Discordant)	NPV (NPV Post Discordant)
BRAF V600E ^1^	8	56	1	2	80.0% (100.0%)	98.2% (100.0%)	88.9% (100.0%)	96.6% (100.0%)
EGFR Exon 19 deletion ^2^	3	62	1	1	75.0% (100.0%)	98.4% (100.0%)	75.0% (100.0%)	98.4% (100.0%)
EGFR L858R ^3^	5	57	3	2	71.4% (100.0%)	95.0% (100.0%)	62.5% (100.0%)	96.6% (100.0%)
EGFR S768I	3	64	0	0	100.0% (100.0%)	100.0% (100.0%)	100.0% (100.0%)	100.0% (100.0%)
EGFR T790M ^4^	3	62	0	2	60.0% (100.0%)	100.0% (100.0%)	100.0% (100.0%)	96.9% (100.0%)
EGFR G719X	2	58	0	0	100.0% (100.0%)	100.0% (100.0%)	100.0% (100.0%)	100.0% (100.0%)
EGFR Exon 20 ^5^ insertion	8	51	1	0	100.0% (100.0%)	98.1% (98.1%)	88.9% (88.9%)	100.0% (100.0%)
EGFR L861Q ^6^	0	59	1	0	N/A (100.0%)	98.3% (100.0%)	N/A (100.0%)	100.0% (100.0%)
ERBB2 insertions ^7^	8	51	1	0	100.0% (100.0%)	98.1% (100.0%)	88.9% (100.0%)	100.0% (100.0%)
KRAS G12C	3	57	0	0	100.0% (100.0%)	100.0% (100.0%)	100.0% (100.0%)	100.0% (100.0%)
ALK fusions ^8^	5	63	0	3	62.5% (71.4%)	100.0% (100.0%)	100.0% (100.0%)	95.5% (97.2%)
MET Exon 14 skipping ^9^	3	66	0	2	60.0% (100.0%)	100.0% (98.9%)	100.0% (80.0%)	97.1% (100.0%)
NTRK1/2/3 fusions ^10^	0	67	0	4	N/A (N/A)	100.0% (100.0%)	N/A (N/A)	94.4% (97.2%)
RET fusions ^11^	3	66	0	2	60.0% (100.0%)	100.0% (100.0%)	100.0% (100.0%)	97.1% (100.0%)
ROS1 fusions ^12^	4	66	1	0	100.0% (100.0%)	98.5% (100.0%)	80.0% (100.0%)	100.0% (100.0%)

^1^ Two BRAF false negatives were not detected by discordant resolution. One BRAF false positive was detected with discordant resolution. ^2^ One EGFR Exon 19 deletion false negative was not detected in discordant resolution; the QC score was below threshold for discordant resolution. One EGFR Exon 19 deletion false positive was detected by discordant resolution. ^3^ Two EGFR L858R false negatives were not detected by discordant resolution. Three EGFR L858R false positives were detected by discordant resolution. ^4^ Two EGFR T790M false negatives were not detected by discordant resolution. ^5^ One EGFR Exon 20 insertion false positive was not detected by discordant resolution. ^6^ One EGFR L861Q false positive was detected by discordant resolution. ^7^ One ERBB false positive was detected by discordant resolution. ^8^ Two ALK false negatives were not detected by discordant resolution. One detected NTRK, novel fusion, but failed to pass the QC threshold. One false negative was detected by discordant resolution and reported as a novel fusion. ^9^ One MET false negative was not detected by discordant resolution. One MET false negative was not sent for discordant resolution. ^10^ Two NTRK false negatives were not detected by discordant resolution. Two NTRK false negatives were detected by discordant resolution but were reported as a novel fusion. ^11^ Two RET false negatives were not detected by discordant resolution. ^12^ One ROS1 false positive was detected by discordant resolution.

**Table 6:** Results of discordant analysis. Results for all discordant samples with comparator and discordant test results. MAFs are reported for all samples where a DNA target was detected. RNA counts, number of positive partitions, are reported for all RNA detections with the ChromaCode panel. MAF (%) and RNA counts are listed as N/A when there was no target detected by the assay. Fusion partners for discordant resolution are reported in Supplementary Table S3.

ID	Comparator Result	Oncomine MAF (%)	ChromaCode Result	Chromacode Estimated MAF (%) (Counts for RNA)	Discordant Result	Discordant MAF (%) or Reads for RNA
1101	ALK	N/A	No Target Detected	N/A	ALK, Novel Isoform	8, 5
1114	NTRK	N/A	No Target Detected	N/A	NTRK, Novel Isoform	29, 12, 12
2031	T790M	91%	L858R, T790M	>80%, 30%	L858R, T790M	69%, 37%
2429	MET	N/A	Not Detected	N/A	No Result	N/A
2720	T790M	0.11%	EGFR L858R	20%	EGFR L858R	90%
2727	EGFR Ex19 Deletion, T790M	14%, 12%	EGFR Ex19 Deletion	44%	EGFR Ex19 Deletion	31%
2733	MET	N/A	Not Detected	N/A	Novel MET Exon fusion (not 14 skipping)	29
3837	No Target Detected	N/A	ROS1	57	ROS1	9
4553	BRAF V600E	N/A	L858R	>80%	L858R	42% ^1^
4570	NTRK	N/A	EGFR Ex19 Deletion	63%	EGFR Ex19 Deletion,	49%
4590	No Target Detected	N/A	BRAF V600E	42%	BRAF V600E	26%
4595	NTRK	N/A	No Target Detected	N/A	NTRK, Novel Isoform	17
5016	NTRK	N/A	No Target Detected	N/A	No Target Detected ^1^	N/A
5143	EGFR EX20 Insertion	0.25%	EGFR EX20 Insertion, L861Q	26%, 7%	EGFR EX20 Insertion, L861Q	22%, 5%
5166	ALK	N/A	Not Detected ^2^	N/A	ALK	22
5386	BRAF V600E	0.26%	EGFR EX20 Insertion	N/A	No Target Detected	N/A
5737	L858R	0.8%	No Target Detected	N/A	No Target Detected	N/A
5739	ALK	N/A	No Target Detected	N/A	Novel NTRK detected ^1^	5
5745	L858R	0.85%	No Target Detected	N/A	No Target Detected	N/A
5894	No Target Detected	N/A	ERBB2	5%	ERBB2	2%
5918	RET	N/A	No Target Detected	N/A	No Target Detected	N/A
7071	ALK, RET	N/A	ALK	21	ALK	35
7334	EGFR Ex19 Deletion	0.2%	No Target Detected	N/A	No Target Detected ^1^	N/A

^1^ Failed sequencing QC. ^2^ Initial run detected ALK; was excluded due to positive plate control failure.

## Data Availability

The raw digital PCR data that support the findings of this study are available from the corresponding author [kcabrera@chromacode.com (accessed on 18 August 2023)] upon reasonable request.

## Supplementary Materials

The following supporting information can be downloaded at: https://www.mdpi.com/article/10.3390/diagnostics13213299/s1, Table S1. Plasmid Insert Sequences. Table S2. FFPE Sample Details. Table S3: Parameters for In Silico Bioinformatic Analysis. Table S4: Results of Empirical Analytical Inclusivity Testing by COSMIC ID for DNA and RNA targets. Table S5: Internal Control performance in clinical samples by sample source vendor. Table S6: Fusion partners for discordant resolution. Table S7. Institutional Review Board Statement. Figure S1: Known MAF vs estimated MAF at varying input. EGFR G719S was spiked in at 2, 5, 10, 20, and 40% known MAF (True_MAF) and plotted against the estimated MAF reported out by ChromaCode Cloud (EGFR G719_maf) at 5 ng, 10 ng, 20 ng, and 40 ng total DNA input. Input amount is defined in ng for each well as measured by Qubit. (*n* = 4 for all MAF/input combinations except 40% known MAF at 40 ng which *n* = 3). Figure S2: Estimated MAF vs input (ng) at varying known MAF. ChromaCode Cloud estimated MAF (EGFR G719_maf) was plotted against input (ng) for each known MAF. Input amount is defined in ng for each well as measured by Qubit. (*n* = 4 for all MAF/input combinations except 40% known MAF at 40 ng which *n* = 3). Figure S3. (A). T790M positive partitions (green), Internal Control (hgDNA) positive partitions (blue), along with calibrator and negative partitions. (B) ROS1 positive partitions (green), Internal Control (hgRNA) positive partitions (blue), along with calibrator and negative partitions.
