# Supplementary material for: Analytical Performance and Concordance with Next-Generation Sequencing of a Rapid, Multiplexed dPCR Panel for the Detection of DNA and RNA Biomarkers in Non-Small-Cell Lung Cancer
Source: Diagnostics (Basel). 2023 Oct 25;13(21):3299. doi: 10.3390/diagnostics13213299 (PMC10650055; doi:10.3390/diagnostics13213299)
Supplement: Supplementary file 1 [file diagnostics-13-03299-s001.zip › diagnostics-2633040-SM.pdf]

Supplementary Materials:

Table S2. FFPE Sample Details.

Sample ID	Oncomine Result	Tissue sampling method	Nucleated Cells per slide	Tissue Surface Area (mm2)	Tumor percent	Age	Gender
C101101	*ALK* Fusion	Lung	35714	75	70	71	M
C101114	*NTRK* Fusion	Lung	83333	150	90	66	M
C408258	*EGFR* exon 19 Deletion	Lung	13333	55	90	59	M
C408259	*EGFR* exon 19 Deletion	Lung	30000	150	100	66	M
C702031	*EGFR* T790M	Lung	25000	105	100	73	F
C702035	*EGFR* L858R	Lung	26316	110	95	65	M
C702040	*KRAS* G12C	Lung	10000	54	100	54	M
XX407334	*EGFR* exon 19 Deletion, *KRAS* G12C	Lung	1000	70	35	72	M
XX411434	*KRAS* G12C	Lung	>5000	143	95	68	M
XX532429	*MET* exon14 skipping	Lung	1000	36	75	72	F
XX662727	*EGFR* exon 19 Deletion, *EGFR* T790M	Lung	>5000	77	70	64	F
XX662730	*ERBB2* exon 20 Insertion	Lung	3000	60	50	60	F
XX662731	*ERBB2* exon 20 Insertion	Lung	1000	40	60	55	M
XX662733	*EGFR* L858R, *MET* exon14 skipping	Lung	2500	48	50	68	F
XX662757	*EGFR* T790M, *EGFR* L858R	Lung	3000	80	80	61	M
XX662765	*ERBB2 e*xon 20 Insertion	Lung	1500	77	30	66	M
XX666465	*EGFR* G719X	Lung	UNKNOWN	UNKNOWN		71	M
XX668154	*BRAF* V600E	Lung	>7500	63	95	75	F
XX702673	*ERBB2* exon 20 Insertion	Lung	3000	84	60	64	M
XX702713	*ERBB2* exon 20 Insertion	Lung	UNKNOWN	UNKNOWN	30	69	F
XX704234	*BRAF* V600E	Lung	1500	100	70	67	M
XX706083	*EGFR* S768I, *EGFR* L858R	Lung	>5000	220	80	41	F
XX739460	*EGFR* exon 20 Insertion	Lung	UNKNOWN	UNKNOWN		35	M
XX739464	*ROS* Fusion	Lung	>5000	72	80	69	M
XX739467	*EGFR* exon 20 Insertion	Lung	UNKNOWN	UNKNOWN		35	M
XX739485	*ROS* Fusion	Lung	>5000	48	80	54	F
XX739596	*RET* Fusion	Lung	>5000	30	95	UNKNOWN	M

XX739627	*EGFR* exon 20 Insertion	Lung	UNKNOWN	UNKNOWN		44	F
XX739629	*ERBB2* exon 20 Insertion	Lung	>5000	78	85	62	F
XX740118	*EGFR e*xon 20 Insertion, *RET* Fusion	Lung	UNKNOWN	UNKNOWN		60	F
XX885918	*RET* Fusion	Lung	2000	81	45	73	M
XX927071	*ALK* Fusion, *RET* Fusion	Lung	2500	117	35	65	M
XY123837	E*GFR* exon 20 Insertion	Lung	2500	72	80	66	M
XY123841	*ROS* Fusion	Lung	2500	98	50	63	M
XY124711	*ALK* Fusion	Lung	>5000	162	60	58	M
XY124734	*ALK* Fusion	Lung	3500	160	50	65	M
XY124735	*ROS* Fusion	Lung	2000	75	50	58	M
XY125737	*EGFR* L858R	Lung	UNKNOWN	UNKNOWN		51	F
XY125739	*ALK* Fusion	Lung	3500	120	80	65	M
XY125745	*EGFR* L858R	Lung	2500	45	80	74	M
XY125888	*MET* exon14 skipping	Lung	UNKNOWN	UNKNOWN		47	M
XY125894	*EGFR* T790M, *EGFR* L858R	Lung	2500	72	50	69	M
XY125903	*ERBB2* Insertion	Lung	2500	119	65	62	M
XY166082	*ERBB2* Insertion	Lung	2500	72	70	68	F
XY204535	*RET* Fusion	Lung	2500	78	50	70	F
XY214570	*NTRK* Fusion	Lung	1500	114	40	58	M
XY214595	*NTRK* Fusion	Lung	>5000	45	90	77	F
XY214643	*EGFR* S768I	Lung	>5000	85	85	67	F
XY214643	*EGFR* G719X	Lung	>5000	85	85	67	F
XY215016	*KRAS* G12C, *NTRK* Fusion	Lung	3500	55	75	68	M
XY215386	*BRAF* V600E	Lung	>5000	110	85	77	M
XY215673	*EGFR* exon 19 Deletion	Lung	>5000	104	75	82	F
XY215683	*RET* Fusion	Lung	3500	99	85	87	M
XY243852	*EGFR* L861Q	Lung	UNKNOWN	UNKNOWN		83	F
XY244180	*EGFR* exon 19 Deletion	Lung	2500	42	55	67	M
XY244238	*ERBB2* Insertion	Lung	2500	30	50	67	F
XY244272	*MET* exon14 skipping	Lung	2500	91	65	66	M
XY244535	*BRAF* V600E	Lung	2500	160	50	33	F
XY244549	*BRAF* V600E	Lung	>5000	99	80	47	M
XY244553	*BRAF* V600E	Lung	>3000	120	90	49	M
XY244557	*BRAF* V600E	Lung	>7500	88	95	52	F
XY244565	*BRAF* V600E	Lung	>10000	112	100	53	M
XY244569	*BRAF* V600E	Lung	>5000	108	90	54	M
XY244572	*ALK* Fusion	Lung	UNKNOWN	UNKNOWN		55	M
XY244580	*ALK* Fusion	Lung	UNKNOWN	UNKNOWN		56	M
XY244590	*EGFR* exon 20 Insertion	Lung	>5001	72	85	58	M

XY244636	*BRAF* V600E	Lung	>5000	77	90	66	M
XY245166	*ALK* Fusion	Lung	UNKNOWN	UNKNOWN		61	M
XY245180	*EGFR* T790M, *EGFR* L858R	Lung	3500	24	50	66	M
XY245225	*ALK* Fusion	Lung	3500	66	65	67	F
XY245233	*ERBB2* Insertion	Lung	>5001	89	80	69	F
XY245263	*EGFR* exon 20 Insertion	Lung	3500	63	85	58	M
XY245278	*MET* exon14 skipping	Lung	3500	91	65	59	F
XY365121	*EGFR* S768I, *EGFR* G719X	Lung	3000	150	80	56	M
XY365134	*EGFR* exon 20 Insertion	Lung	UNKNOWN	UNKNOWN		62	M
XY365143	*EGFR* exon 20 Insertion	Lung	3000	100	60	59	M
XX662720	*EGFR* T790M	Lung	UNKNOWN	UNKNOWN		63	M

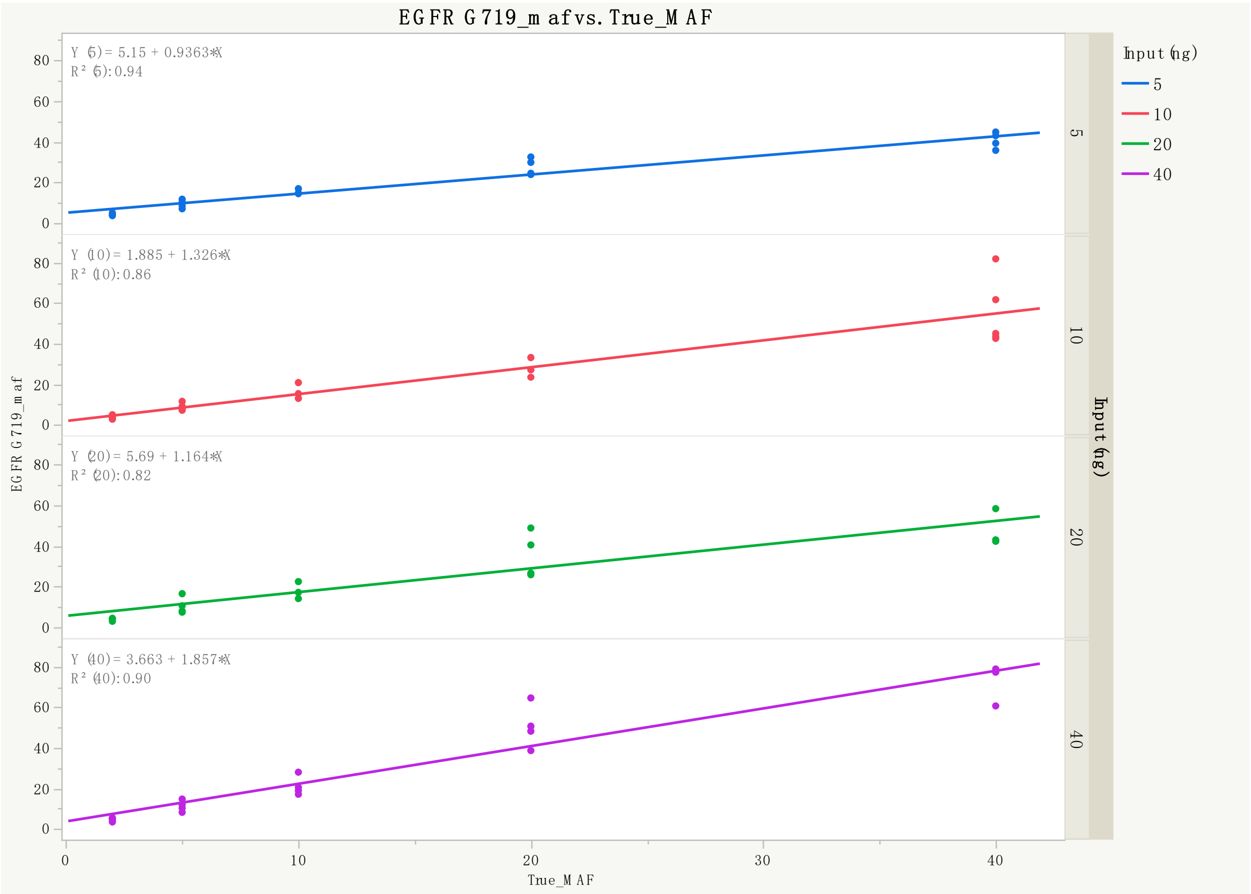

Figure S1: Known MAF vs estimated MAF at varying input. *EGFR* G719S was spiked in at 2, 5, 10, 20, and 40% known MAF (True_MAF) and plotted against the estimated MAF reported out by ChromaCode Cloud (*EGFR* G719_maf) at 5 ng, 10 ng, 20 ng, and 40 ng total DNA input. Input amount is defined in ng for each well as measured by Qubit. (n = 4 for all MAF/input combinations except 40% known MAF at 40 ng which n = 3)

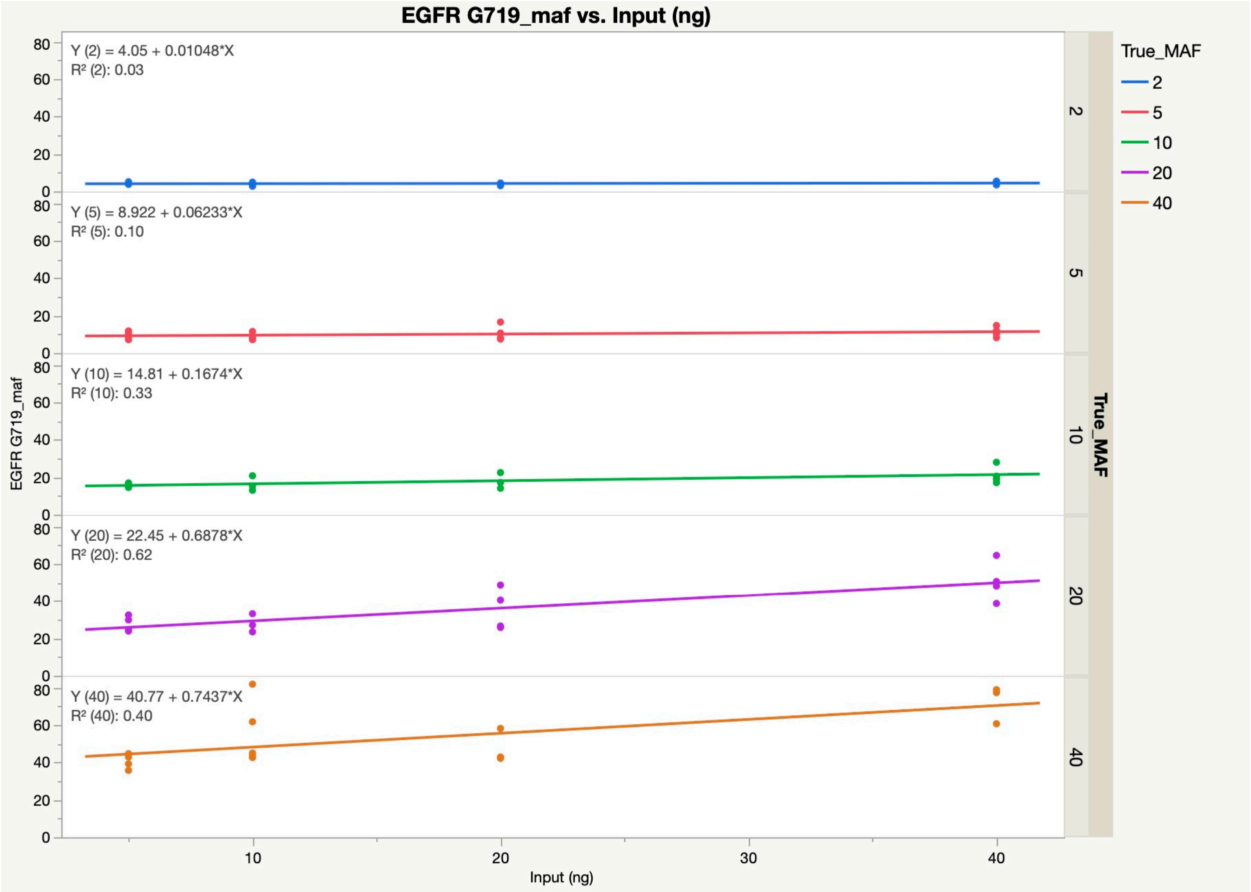

Figure S2: Estimated MAF vs input (ng) at varying known MAF. ChromaCode Cloud estimated MAF (EGFR G719_maf) was plotted against input (ng) for each known MAF. Input amount is defined in ng for each well as measured by Qubit. (n = 4 for all MAF/input combinations except 40% known MAF at 40 ng which n = 3)

Table S3: Parameters for In Silico Bioinformatic Analysis.

**DNA Variants**		
**Variable**	**Input**	
CosmicMutantExport.tsv.gz Version v97 Download Date 08FEB2023		
Primary site	Lung	
Histology subtype	%in%c("adenocarcinoma", "non_small_cell_carcinoma", "squamous_cell_carcinoma", "large_cell_carcinoma", "large_cell_neuroendocrine_carcinoma", "bronchioloalveolar_adenocarcinoma", "acinar_adenocarcinoma", "sarcomatoid_carcinoma")	
GRCh	38	
Mutation somatic status	`%in%c("Reported in another cancer sample as somatic","Confirmed somatic variant")	
**Filtering by Variant Type**		
**Variant Type**	**Filter Column**	**Input**
EGFR exon 19 deletion	HGNC ID	3236 (EGFR)
	Mutation CDS	contains c.2185-c.2283
	Mutation Description	contains "Deletion", ignore.case=TRUE
EGFR exon 20 insertion	HGNC ID	3236 (EGFR)
	Mutation CDS	contains c.2284-c.2469
	Mutation Description	contains "Insertion", ignore.case=TRUE
ERBB2 exon 20 insertion	HGNC ID	3430 (ERBB2)
	Mutation CDS	contains c.2308-c.2493
	Mutation Description	contains "Insertion", ignore.case=TRUE
**RNA Fusions**		
**Variable**	**Input**	
CosmicFusionExport.tsv.gz Version v97 Download Date 08FEB2023		
Primary site	Lung	

Histology subtype	%in%c("adenocarcinoma", "non_small_cell_carcinoma", "squamous_cell_carcinoma", "large_cell_carcinoma", "large_cell_neuroendocrine_carcinoma", "bronchioloalveolar_adenocarcinoma", "acinar_adenocarcinoma", "sarcomatoid_carcinoma")

Filtering by Individual Fusion Type

**Fusion Type**	**Filter**	**Input**
ALK fusion subset of NSCLC fusion data	3'_GENE_NAME	ALK
RET fusion subset of NSCLC fusion data	3'_GENE_NAME	RET
ROS1 fusion subset of NSCLC fusion data	3'_GENE_NAME	ROS1
NTRK1 fusion subset of CosmicFusionExport*	3'_GENE_NAME	NTRK1
NTRK2 fusion subset of CosmicFusionExport*	3'_GENE_NAME	NTRK2
NTRK3 fusion subset of CosmicFusionExport*	3'_GENE_NAME	NTRK3

Table S4: Results of Empirical Analytical Inclusivity Testing by COSMIC ID for DNA and RNA targets.

	Target	Cosmic ID	Detected
1	ALK	COSF1051	2/2
2	ALK	COSF1056	2/2
3	ALK	COSF1058	2/2
4	ALK	COSF1060	2/2
5	ALK	COSF1064	2/2
6	ALK	COSF1127	2/2
7	ALK	COSF1257	2/2
8	ALK	COSF1264	2/2
9	ALK	COSF1276	2/2
10	ALK	COSF1296	2/2
11	ALK	COSF1300	2/2
12	ALK	COSF1381	2/2
13	ALK	COSF1430	2/2
14	ALK	COSF1461	2/2
15	ALK	COSF1541	2/2
16	ALK	COSF1544	2/2
17	ALK	COSF1612	2/2
18	ALK	COSF1614	2/2
19	ALK	COSF1616	2/2
20	ALK	COSF1620	2/2
21	ALK	COSF1625	2/2
22	ALK	COSF1712	2/2
23	ALK	COSF409	2/2
24	ALK	COSF413	0/2
25	ALK	COSF415	2/2
26	ALK	COSF421	2/2
27	ALK	COSF424	2/2
28	ALK	COSF426	2/2
29	ALK	COSF428	2/2
30	ALK	COSF434	2/2
31	ALK	COSF437	2/2
32	ALK	COSF439	2/2
33	ALK	COSF441	2/2
34	ALK	COSF444	2/2
35	ALK	COSF459	2/2
36	ALK	COSF460	2/2
37	ALK	COSF469	2/2
38	ALK	COSF477	2/2
39	ALK	COSF478	2/2
40	ALK	COSF487	2/2
41	ALK	COSF732	2/2

42	EGFR Del	COSM12370	2/2
43	EGFR Del	COSM12382	2/2
44	EGFR Del	COSM12383	2/2
45	EGFR Del	COSM12384	2/2
46	EGFR Del	COSM12422	2/2
47	EGFR Del	COSM12678	2/2
48	EGFR Del	COSM13556	2/2
49	EGFR Del	COSM24267	0/2
50	EGFR Del	COSM26038	2/2
51	EGFR Del	COSM26704	0/2
52	EGFR Del	COSM26718	2/2
53	EGFR Del	COSM28517	2/2
54	EGFR Del	COSM6218	2/2
55	EGFR Del	COSM6225	2/2
56	EGFR Del	COSM6255	2/2
57	EGFR Del	COSM6256	2/2
58	EGFR Ins	COSM12376	2/2
59	EGFR Ins	COSM12378	2/2
60	EGFR Ins	COSM12380	2/2
61	EGFR Ins	COSM12381	2/2
62	EGFR Ins	COSM12427	0/2
63	EGFR Ins	COSM13428	2/2
64	EGFR Ins	COSM18432	2/2
65	EGFR Ins	COSM26720	0/2
66	ERRB2	COSM12552	2/2
67	ERRB2	COSM12553	0/2
68	ERRB2	COSM12558	2/2
69	ERRB2	COSM26681	2/2
70	ERRB2	COSM6438151	0/2
71	G719X	COSM6252	2/2
72	G719X	COSM6253	2/2
73	NTRK	COSF1323	2/2
74	NTRK	COSF1324	2/2
75	NTRK	COSF1326	2/2
76	NTRK	COSF1329	2/2
77	NTRK	COSF1446	2/2
78	NTRK	COSF1448	1/2
79	NTRK	COSF1534	2/2
80	NTRK	COSF1536	2/2
81	NTRK	COSF1653	2/2
82	NTRK	COSF823	2/2
83	RET	COSF1230	2/2
84	RET	COSF1232	2/2
85	RET	COSF1234	2/2

86	RET	COSF1236	2/2
87	RET	COSF1253	2/2
88	RET	COSF1262	2/2
89	RET	COSF1340	2/2
90	RET	COSF1481	2/2
91	RET	COSF1491	2/2
92	RET	COSF1498	2/2
93	RET	COSF1503	2/2
94	RET	COSF1507	2/2
95	RET	COSF1509	2/2
96	RET	COSF1511	2/2
97	RET	COSF1513	2/2
98	RET	COSF1515	2/2
99	RET	COSF1519	2/2
100	RET	COSF1521	2/2
101	RET	COSF1525	2/2
102	RET	COSF1687	2/2
103	ROS1	COSF1139	2/2
104	ROS1	COSF1188	2/2
105	ROS1	COSF1196	2/2
106	ROS1	COSF1198	2/2
107	ROS1	COSF1198	2/2
108	ROS1	COSF1202	2/2
109	ROS1	COSF1259	2/2
110	ROS1	COSF1261	2/2
111	ROS1	COSF1265	2/2
112	ROS1	COSF1267	2/2
113	ROS1	COSF1269	2/2
114	ROS1	COSF1273	2/2
115	ROS1	COSF1278	2/2
116	ROS1	COSF1280	2/2
117	ROS1	COSF1478	2/2
118	ROS1	COSF1627	2/2
119	ROS1	COSF1631	2/2
120	ROS1	COSF1637	2/2
121	ROS1	COSF1639	2/2
122	ROS1	COSF1641	2/2
123	ROS1	COSF1643	2/2
124	ROS1	COSF1645	2/2
125	ROS1	COSF1647	2/2
126	ROS1	COSF1663	2/2
127	ROS1	COSF1671	2/2
128	ROS1	COSF1673	2/2

Table S5: Internal Control performance in clinical samples by sample source vendor.

Vendor	All Wells Pass	1+ Well IC Failure	% with 1 Failure
Vendor A	3	5	63%
Vendor B	29	0	0%
Vendor C	53	16	23%

Table S6: Fusion partners for discordant resolution.

ID	Fusion Coordinates
1101	chr2:299621113, chr2:29446553<br>chr2:29498458, chr2:29498378
1114	chr18:45085964, chr1:156851219<br>chr17:9138266, chr9:156851219<br>chr17:9138266, chr9:87475930
3837	chr6:117659858, chr6:117658503
5739	chr1:156849914, chr1:156851249
4595	chr9:87367700, chr9:87356807<br>chr9:87367000: chr9:87317074<br>chr9:87285856: chr9:87317138
7566	chr6:117723136, chr6117718279

S3. 2-D plots of representative dPCR data generated during the Analytical Sensitivity study.

Figure S3A. T790M positive partitions (green), Internal Control (hgDNA) positive partitions (blue), along with calibrator and negative partitions.

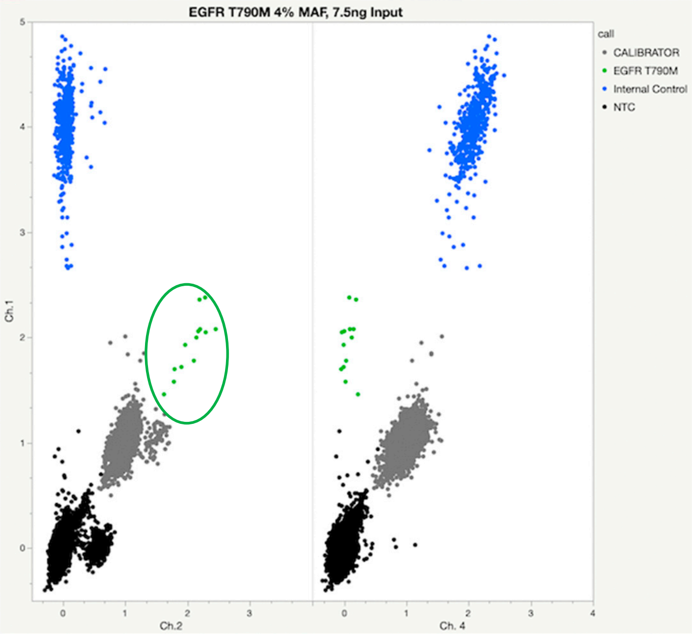

Figure S3B. ROS1 positive partitions (green), Internal Control (hgRNA) positive partitions (blue), along with calibrator and negative partitions.

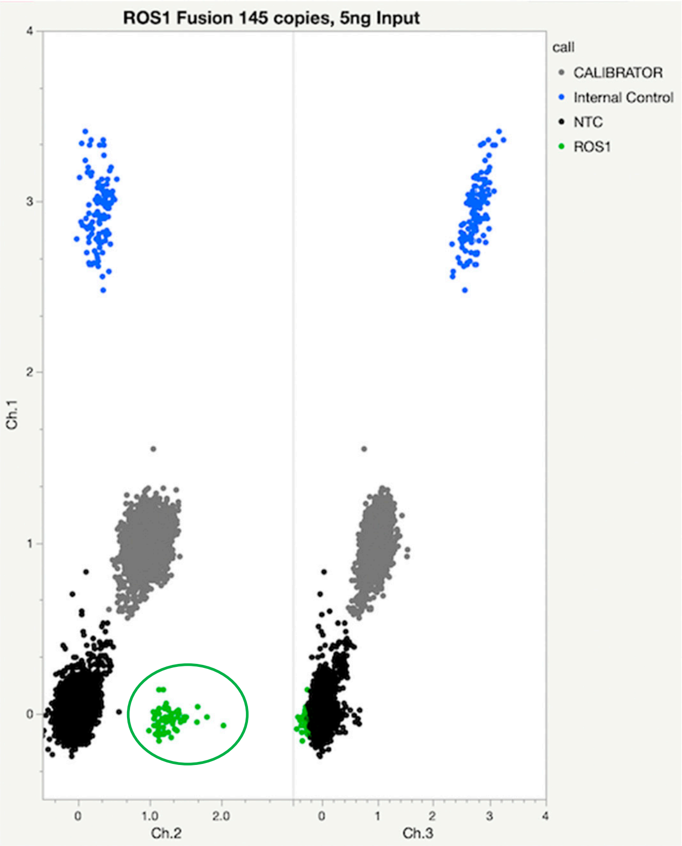

Table S7. Institutional Review Board Statement.

IRB/Ethic Committee Name	Protocol/IRB #	Approval Date
BioChain Institute Inc. IRB #1 - Biomedical	IRB00008283	6/9/21
Vanderbilt University IRB	IRB 010294	10/15/22
Advarra IRB	CR00425931	3/6/23
	Pro00051469	3/6/23
